# Supplementary material for: Tunable Octdong and Spindle-Torus Fermi Surfaces in Kramers Nodal Line Metals
Source: Nat Commun. 2025 Dec 12;16:11128. doi: 10.1038/s41467-025-66284-9 (PMC12705649; doi:10.1038/s41467-025-66284-9)
Supplement: Supplementary file 1 — Supplementary Information [file 41467_2025_66284_MOESM1_ESM.pdf]

Tunable Octdong and Spindle-Torus Fermi Surfaces in Kramers Nodal Line Metals

Gabriele Domaine[1,2], Moritz M. Hirschmann[2,3], Kirill Parshukov[2], Mihir Date[1,4], Holger L. Meyerheim[1], Matthew D. Watson[4], Katayoon Mohseni[1], Sydney K. Y. Dufresne[1], Shigemi Terakawa[1,5,6], Marcin Rosmus[7,8], Natalia Olszowska[7], Stuart S. P. Parkin[1], Andreas P. Schnyder[2†], and Niels B. M. Schröter[1†*]

[1]Max Planck Institut für Mikrostrukturphysik, Weinberg 2, 06120 Halle, Germany

[2]Max-Planck-Institut für Festkörperforschung, Heisenbergstrasse 1, D-70569 Stuttgart, Germany

[3]RIKEN Center for Emergent Matter Science, Wako, Saitama 351-0198, Japan

[4]Diamond Light Source Ltd, Harwell Science and Innovation Campus, Didcot, OX11 0DE, United Kingdom

[5]Department of Applied Physics, Graduate School of Engineering, The University of Osaka, Osaka 565-0871, Japan

[6]Center for Future Innovation, Graduate School of Engineering, Osaka University, Osaka 565-0871, Japan

[7]SOLARIS National Synchrotron Radiation Centre, Jagiellonian University, Krakow, Poland and

[8]Université Paris-Saclay, CNRS, Institut des Sciences Moléculaires d'Orsay, 91405, Orsay, France

(Dated: October 27, 2025)

† These authors jointly supervised this work.
* niels.schroeter@mpi-halle.mpg.de

SUPPLEMENTARY MATERIALS

A. Almost movable nodal lines

Almost movable nodal lines (AMNLs) are enforced by crystal symmetries [1], e.g., by those of the 3R phase of TaS_2. AMNLs are KNLs [2] that are only pinned to TRIMs, but not to high-symmetry paths. Inside the mirror plane, such a line degeneracy connecting TRIMs can have an arbitrary shape as long as the global connectivity is respected [see Fig. 6a-b in the main text].

We give the comprehensive classification of space groups enforcing AMNLs in Table I [3]. For the AMNL space groups listed in Table I, we also note all other symmetry-enforced band crossings in the presence of SOC, including twofold (Weyl) and fourfold points, pinned nodal lines, and movable hour-glass nodal lines which are denoted following the notation of Ref. [1, 4]. AMNL and movable hour-glass nodal lines are denoted by a bracket listing TRIMs and/or degenerate lines on the mirror plane separated by a semicolon. At the TRIMs before (after) the semicolon different (identical) mirror eigenvalues are paired by the space group symmetries, e.g., time-reversal symmetry for the AMNL. By the argument provided for the existence of AMNL [1], each TRIM on the left side of the semicolon in this notation is part of a nodal line, e.g., for space group (SG) 6 Pm the bracket (Γ,B,Y,A;–) all TRIMs within the mirror plane are part of AMNLs. This is generally true, but for not listed SGs the nodal line passing through such TRIMs is pinned instead of AMNL.

On any path within the Brillouin zone connecting points with the same mirror eigenvalue pairing, the number of nodal lines must be even, so in the simplest case zero. Between points with different pairing, there have to be an odd number of nodal lines, to provide the necessary exchange of mirror eigenvalues. The latter implies the presence of hour-glass nodal lines. Notably, AMNL and hour-glass nodal lines reside within different (direct) band gaps, AMNL occur within all odd numbered gaps, $2n+1,\ n \in \mathbb{N}_0$ sorted in energy, whereas hour-glass nodal lines occur within every second even numbered gap, $2+4n$. Notably, some TRIMS exhibit AMNL depending on the orbital character of the bands, in other words, the realized symmetry representation. In these cases, a pinned nodal line can but does not always exist that crosses a TRIM and replaced the AMNL, we have marked the TRIMs and pinned nodal lines with a star in Table I.

SUPPLEMENTARY TABLE I. Almost movable nodal lines. All points are given in the Bilbao Crystallographic Server (BCS) [5] notation. Other lines are taken from Refs. [1, 4] for tetragonal and orthorhombic SGs, otherwise from the BCS. Mark * indicates that the existence of the topological crossing depends on the representation. Notation $(\Gamma_i; -)$ represents the fact that the AMNL passes Γ_i TRIM. Numbers in brackets show the degeneracy at high-symmetry points and lines. $(A; B)$ represents the hourglass nodal line between degenerate points (or lines) [1, 4].

SG	AMNL position	points	other lines	comment
6 Pm	(Γ,B,Y,A;–) (Z,C,D,E;–)			
7 Pc	(Γ,Y;–) (Z,C;–)		(Γ,Y;B,A), (Z,C;D,E), BD, AE	
8 Cm	(Γ,Y,A,M;–)	L(2),V(2)		
9 Cc	(Γ,Y;–)	L(2),V(2)	(Γ,Y;A,M), AM	
38 Amm2	(S;–) (R;–)		ΓY, ZA	
40 Ama2	(S;–) (R;–)		(ΓY;Z,T), ZT	
44 Imm2	(S;–) (R;–)	T(2)	ΓX	
46 Ima2	(S;–)	T(2)	RW, (ΓX;R)	
107 I4mm	(N;–)		ΓM, PX	
109 $I4_1md$	(N;–) (P*;–)	M(4)	(ΓM;X), XM	AMNLs at P appear for a particular 2-fold rotation eigenvalue
110 $I4_1cd$	(P;–)	M(4)	(ΓM;N)(4), (XM;P)(4)	
119 I-4m2	(N;–)		ΓM	
156 P3m1	(M,L,Γ*,A*;–)		ΓA*	Depending on ΓA rep, pinned NL or 3 AMNLs in 3 mirror planes at Γ, A
157 P31m	(M,L,Γ*,A*;–)		ΓA*, KH*	Depending on ΓA rep, pinned NL or 3 AMNLs in 3 mirror planes at Γ, A; NL at KH appears for a particular rep
158 P3c1	(M,Γ*;–)	A(4)*	(Γ,M;A,L), AH, LH, ΓA*	Depending on ΓA rep, pinned NL or 3 AMNLs in 3 mirror planes at Γ
159 P31c	(M,Γ*;–)	A(4)*	(Γ,M;A,L), AL, KH*, ΓA*	Depending on ΓA rep, pinned NL or 3 AMNLs in 3 mirror planes at Γ

160 R3m	(L,F,Γ*,T*;–)		ΓT*, P*	Depending on ΓT rep, pinned NL or 3 AMNLs in 3 mirror planes at Γ, T; P is symmetric under mirror and 3-fold rotational symmetries, the NL appears for a particular rep
161 R3c	(F,Γ*;–)	T(4)*	(Γ,F;T,L), ΓT*, P*, Y, B	Depending on ΓT rep, pinned NL or 3 AMNLs in 3 mirror planes at Γ; P is symmetric under mirror and 3-fold rotational symmetries, the NL appears for a particular rep; Y, B are the $\mathcal{TM}$-symmetric lines
174 P-6	(Γ,M;–) (A,L;–)		ΓA*	At Γ, A 3 AMNL intersect
216 F-43m	(L;–)*	Γ(4)*	ΓX, ΓL*	Depending on ΓL rep, pinned NL or 3 AMNLs in 3 mirror planes at L

B. Spatial map

We use the distinction in the number of bands between 2H and 3R polytypes along the G-M direction to distinguish the two phases when scanning the photon beam over the sample. This process is illustrated in Fig. 1.

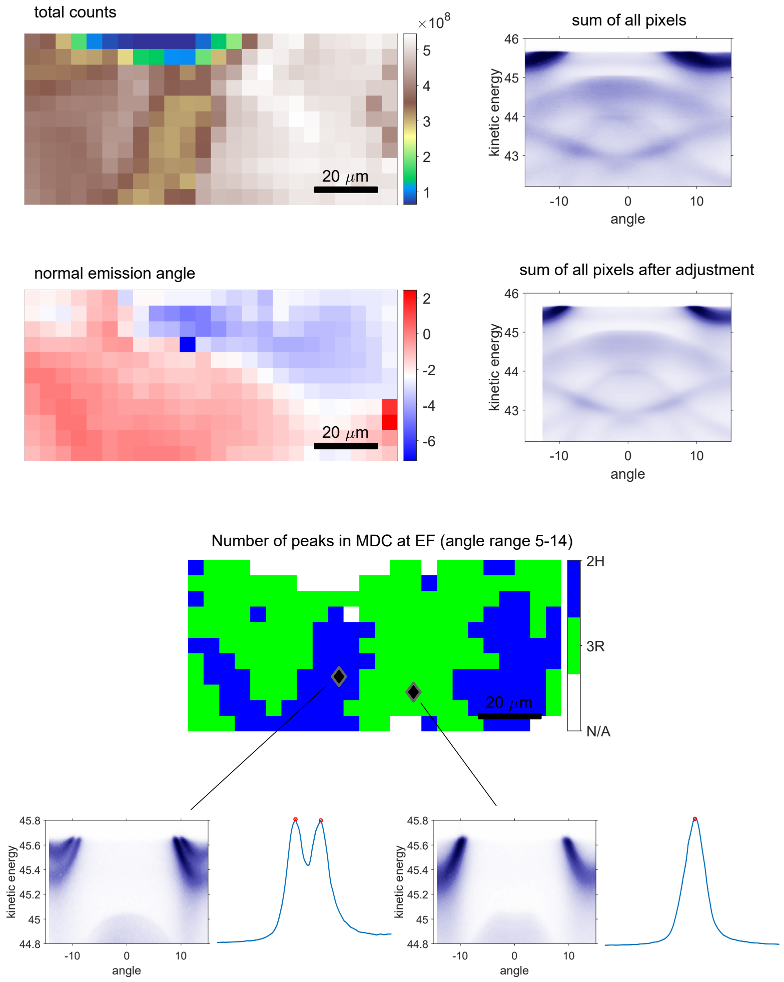

SUPPLEMENTARY FIG. 1. **Extracting domain of different polytypes from ARPES data.** Since TaS_2 is a flaky sample, the normal emission angle varies substantially in a raster scan of the sample. To account for this, we first apply a routine to automatically determine the normal emission angle at each pixel of the spatial map, using an autocorrelation function, and then adjust the angle scale of each pixel so normal emission is at 0. We then extract MDCs in the angle range 5-14 degrees, and apply a peakfinding algorithm to the MDC. In case one peak is found, we assign this pixel to the 3R phase, in case 2 peaks are found we assign the 2H phase, and in some regions where mutiple peaks are found (due to flakes or noise or low counts) we assign N/A.

C. Matrix elements effects

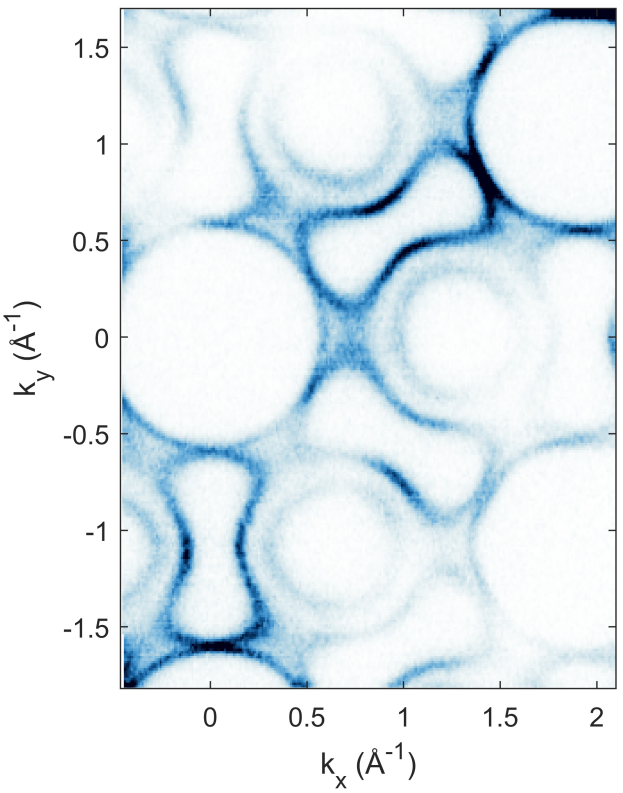

SUPPLEMENTARY FIG. 2. **Fermi surface map of the 3R phase measured with hν = 126 eV photons.** We can see that even in the neighboring Brillouin zones, where the matrix elements are different, the hole and electron pockets are connected at one point.

D. XRD peak analysis

For the two fitted peaks for the 3R phase in Fig. 2c we obtain a Full-Width at Half Maximum (FWHM) of approximately 0.04 radians. Using the Scherrer equation we estimate an approximate thickness of 37 Å. Given a lattice parameter $c \approx 18$ Å, we get approximately two unit cells, which correspond to 6 1H monolayers, in good agreement with the 5 quantized bands that we see from ARPES and report in Fig. 4 in the main text.

In order to quantitatively analyze the crystal structures of the 2H and the 3R phase being simultaneously present in the sample we have carried out quantitative x-ray diffraction experiments using our in-house metal jet (Ga) x-ray source ($\lambda = 1.341\mathring{A}$) and a custom made six-circle diffractometer equipped with a two-dimensional pixel detector [6]. Integrated intensities were collected for a given reflection (HKL) by transverse θ-scans. In this way we collected about 40-50 reflection intensities for each sample which were subsequently averaged to 19 symmetry independent ones using point group symmetry 3m and 6mm for the 2H and the 3R phase, respectively. Using the squared structure factor magnitudes $[|F(obs)|2]$ derived by multiplying instrumental correction factors by the intensities[7, 8]. The structural refinement was carried out by the program "Shelx" [9] using published structural models (see below) as initial models [10, 11].

For both data sets we could achieve very good fits. The fit quality is usually characterized by the wR2, the Goodness of Fit (GOF) parameter and the unweighted residuum (Ru) [9, 12] . Generally, we find GOF values somewhat below 1.0 and Ru well below 0.1. Tables T1 to T4 list the refined structural parameters and compares the calculated and observed squared structure factor magnitudes for the 2H and 3R phase. The structural parameters are in good agreement with previously published data [10, 11] (see below for a more detailed discussion). We emphasize that the data sets were collected immediately one after another using the same primary intensity and diffractometer geometry. Thus, the overall scaling of the $|F_{\mathrm{obs}}|^2$ allows to estimate the relative abundance of the two phases. We obtain overall scaling factors (OSF) of 0.183±0.014 and 0.024±0.002, for the 2H and the 3R phase respectively, i.e. they differ by a factor 7.6. Since the OSF is the number by which the $|F_{\mathrm{obs}}|^2$ are divided to fit to the $|F_{\mathrm{calc}}|^2$, it can be concluded that the volume ratio between the 2H- and the 3R-phase in the sample is equal to approximately 8.

Our model is in close agreement with that of Gotoh et al. However, in our analysis we find some additional details. This is outlined using the Figure S1 showing our structural model viewed along the $[11\bar{1}0]$ direction. Only two out of three S-Ta-S sheets composing the unit cells are shown for simplicity. The Ta1 atoms are located within an octahedrally coordinated site between the two hexagonal sheets of S atoms (S1 and S2), while there are two (instead of one in Ref. [11]) "interstitial" sites which are labelled by Ta2 and Ta3, respectively. These atoms are located near the top and bottom surface of the hexagonal packed sulfur sheets in a threefold hollow site. The site occupancy factors (SOF) of Ta2 and Ta3 site are about 0.1, while for Ta1 we find SOF=0.8 (instead of 1.0 in Ref. [11]). Uncertainties for SOF is in the

SUPPLEMENTARY TABLE II. List of calculated and observed squared structure factor amplitudes for the 2H phase. The agreement parameters are $wR_2 = 0.221$, GOF = 1.131, $R_u = 0.078$ for all 19 reflections. The overall scale factor OSF = OSF = 0.1831 ± 0.0140.

H	K	L	$\|F_{\text{calc}}\|^2$	$\|F_{\text{obs}}\|^2$	$\sigma[\|F_{\text{obs}}\|^2]$
0	1	1	825	815	107
0	2	1	406	356	47
-1	3	1	295	244	32
0	1	2	9098	8763	1157
0	2	2	3725	3133	413
-1	3	2	1743	1802	237
0	1	3	703	1394	184
0	2	3	388	558	73
-1	3	3	287	360	47
0	1	4	8804	7203	951
0	2	4	4046	3869	510
-1	3	4	2164	2194	289
0	1	5	431	465	61
0	2	5	276	297	39
-1	3	5	209	206	27
0	1	6	2458	1951	258
0	2	6	1095	1360	179
0	2	7	251	187	24
-1	2	8	2163	2771	378

SUPPLEMENTARY TABLE III. List of structural parameters for TaS2-2H. Atomic coordinates are given in relative units of the trigonal unit cell (space group Nr. 194 P63/mmc) with a=b=3.314 Å c=12.097 Å. Parameters labelled by an asterisk are fixed by symmetry. The site occupancy factor (SOF) has been fixed to 1 for both sites.

Atom	Site	x	y	z	SOF	U ($\AA^2$)
S1	$4f$	$\frac{1}{3}^*$	$\frac{2}{3}^*$	0.124(2)	1.0	0.012(7)
Ta1	$2b$	0^*	0^*	0.250^*	1.0	0.058(9)

0.05 range. In total, this indicates that the crystal structure is close to perfect stoichiometry. We finally emphasize that the consideration of the two Ta sites is mandatory for achieving a good fit. For instance, for the stoichiometric model with no interstitial we obtain agreement parameters of GOF=1.17, Ru=0.124 and wR2=0.2927, for the model considering only Ta1, the parameters GOF=0.99, Ru=0.1012 and wR2=0.2394 are obtained, which are at least

SUPPLEMENTARY TABLE IV. List of calculated and observed squared structure factor amplitudes for the 3R phase. Agreement parameters: $wR_2 = 0.205$, GOF = 0.882, $R_u = 0.078$ for all 19 reflections. Overall scale factor OSF = 0.0236 ± 0.002.

H	K	L	$\vert F_{\mathrm{calc}}\vert^2$	$\vert F_{\mathrm{obs}}\vert^2$	$\sigma[\vert F_{\mathrm{obs}}\vert^2]$
-1	1	-11	830	756	162
-2	2	-10	1016	774	160
-1	1	-8	5653	4662	1008
-2	3	-8	2874	3582	792
-2	2	-7	6679	5850	1278
-1	1	-5	15716	14526	3168
-2	3	-5	7338	7714	1674
-2	2	-4	9402	7740	1655
-1	1	-2	9642	8766	1908
-2	2	-1	4310	4212	918
-1	1	1	7622	10620	2340
-2	3	1	3504	3222	702
-2	3	4	6634	7956	1746
-2	2	5	10485	11142	2448
-1	1	7	9545	12816	2826
-2	3	7	4739	4734	1044
-2	2	8	4001	4248	936
-1	1	10	1521	3060	666
-2	2	11	535	594	126

SUPPLEMENTARY TABLE V. Structural parameters for TaS_2-3R. Atomic coordinates are given in relative units of the trigonal unit cell]space group Nr. 160 (R3m)] with $a = b = 3.32$ Å, $c = 18.29$ Å. Parameters labeled by an asterisk are fixed by symmetry. The uncertainty of the SOF's is in the 0.05–0.10 range. Data are compared with those of Gotoh et al. [11] in Table VI.

Atom	Site	x	y	z	SOF	U (Å^2)
S1	$3a$	0^*	0^*	0.252(11)	1.0	0.053(14)
S2	$3a$	0^*	0^*	0.417(7)	1.0	0.053(14)
Ta1	$3a$	0^*	0^*	0.*	0.8	0.053(14)
Ta2	$3a$	0^*	0^*	0.739(18)	0.1	0.053(14)
Ta3	$3a$	$\frac{1}{3}^*$	$\frac{2}{3}^*$	0.265(14)	0.1	0.053(14)

SUPPLEMENTARY TABLE VI. Structural parameters for TaS_2-3R published by Gotoh et al. [11].

Atom	Site	x	y	z	SOF	U ($\AA^2$)
S1	$3a$	0^*	0^*	0.2484(8)	1.0	0.008(2)
S2	$3a$	0^*	0^*	0.4220(8)	1.0	0.100(2)
Ta1	$3a$	0^*	0^*	$0.^*$	1.0	0.004(1)
Ta2	$3a$	0^*	0^*	0.818(18)	0.8(1)	0.010(6)

20% enhanced as compared with the best fit values.

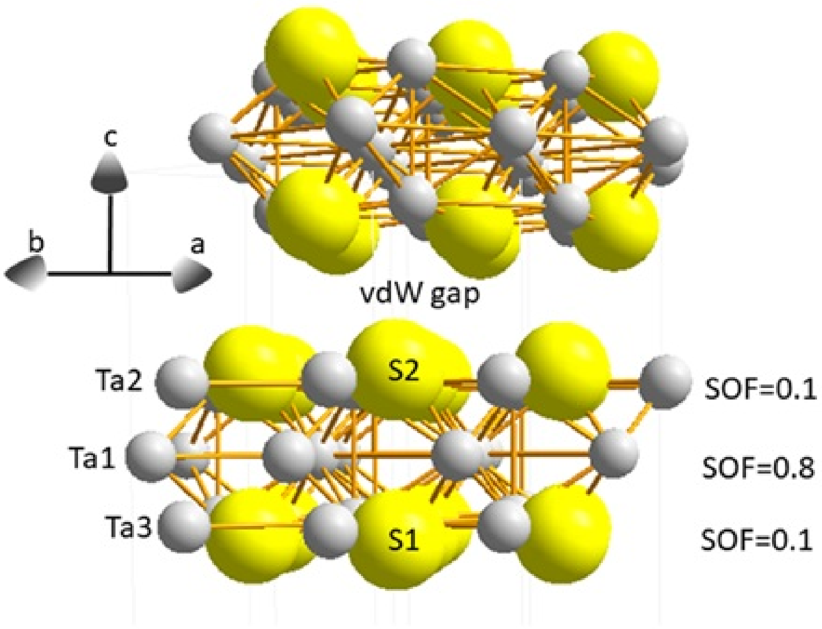

SUPPLEMENTARY FIG. 3. Model of the TaS_2-3R structure derived from our analysis. Atom labels refer to Table V. We find two interstitial atoms, Ta2 and Ta3, located above and below the hexagonal sulfur sheets (S1, S2).

E. DFT and TB model comparison

To achieve reasonable agreement with DFT, we construct a tight-binding model comprising 17 hopping parameters, including terms up to fifth-nearest-neighbor interactions. While some discrepancies are expected with respect to the DFT calculations due to the limitations of a minimal two-band model, particularly its inability to capture hybridization with adjacent bands, the overall connectivity of the nodal lines observed in DFT is qualitatively reproduced.

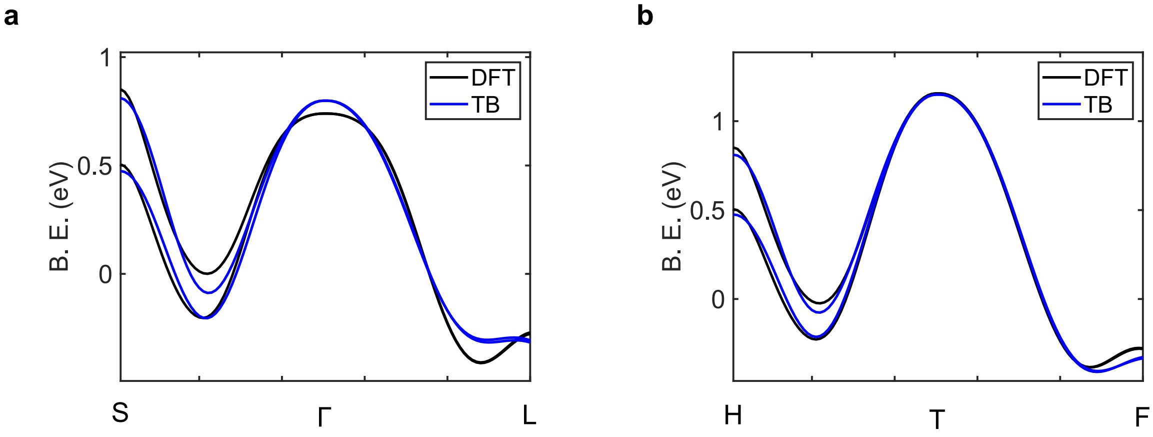

SUPPLEMENTARY FIG. 4. **Agreement between DFT and TB calculations.** Comparison of the DFT (black) and tight-binding (blue) band structures along different high-symmetry paths.

F. Quantization in the 2H phase

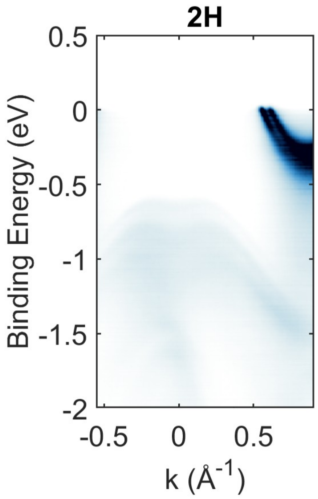

SUPPLEMENTARY FIG. 5. **Quantization of the valence band in the 2H phase.** Just as in the 3R phase, certain areas of the 2H phase exhibit a quantization of the valence band.

G. Identification of TaS_2 polytypes with ARPES

TMDCs can crystallize into various polytypic structures [13]. In the well studied 2H-TaS_2 phase, two doubly degenerate bands are crossing the Fermi level along the Γ-M direction, which are split due to interlayer interaction, and exist due to two metal atoms per unit cell (Fig. 2b-e in the main text). In contrast, the 3R-TaS_2 phase, there is only a single band along the Γ-M that is weakly split by spin-orbit coupling due to only one metal atom per unit cell and the absence of interlayer splitting, which is consistent with the ARPES band dispersion shown in Fig. 2i in the main text. The only other experimentally realized polytype with a single atom per unit cell is 1T-TaS_2. Since this phase has inversion symmetry, it would not show the spin-orbit coupling induced band splitting along the M-K direction that is clearly observed in Fig. 2g in the main text. The observed Fermi-surface in Fig. 2i in the main text must therefore originate from the 3R-TaS_2 phase. It should also be noted that the valence band (as seen in Fig. 4a in the main text) shows at least two quantized levels. This suggests that the system is not a single 1H mono-layer. Furthermore, the two fundamental building blocks of the TaS_2 TMDCs are the 1T and 1H monolayers, where the Ta atom is in octahedral and trigonal prismatic coordination, respectively. These two coordinations lead to significant differences in the energy of the Ta core levels. Since we do not observe any difference in our two phases (Supplementary Fig. 6), we can exclude all polytypes involving octahedral coordination sites. This leaves us with only the 2H (a, b, and c), 3R, and 4H (a and c) polytypes. Of these, only the 3R polytype has a single Ta site in the primitive unit cell, leading to a single band at the Fermi level.

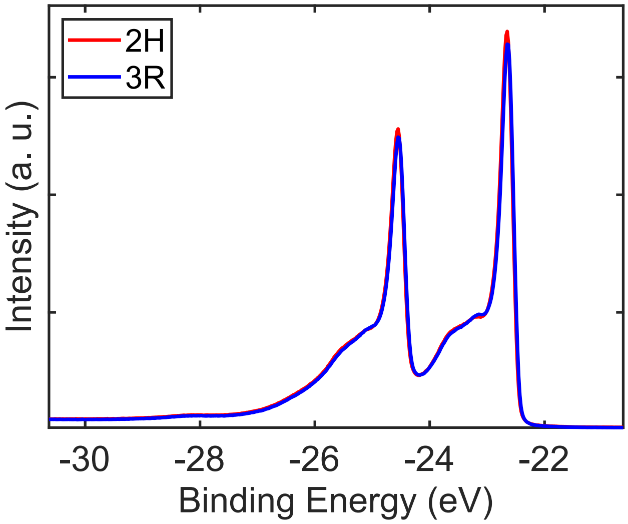

SUPPLEMENTARY FIG. 6. **Tantalum 4f core levels.** The core levels of a 2H patch and a 3R patch showing almost identical features, suggesting the presence of a single type of coordination of the Ta atoms. Notice that the core levels for the 1T polytype, as well as other polytypes including 1T monolayers exhibit a different number of core levels peaks with different relative intensities [14, 15].

H. 3R phase in another sample

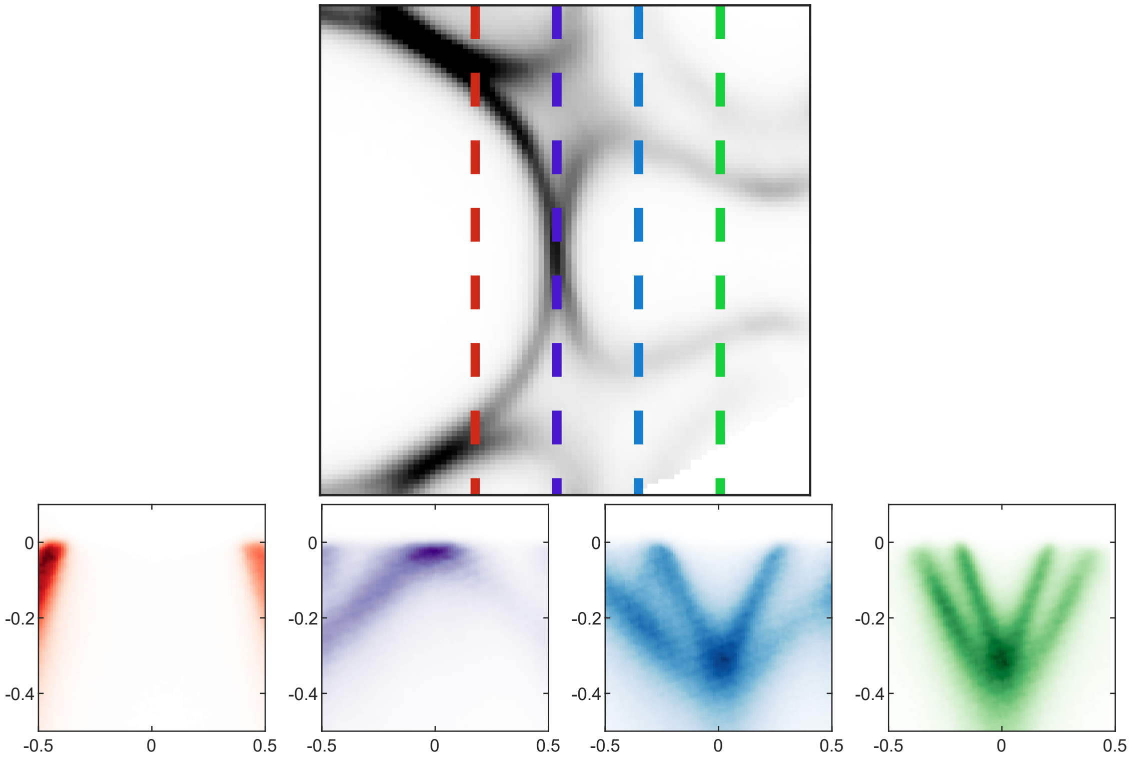

SUPPLEMENTARY FIG. 7. **Nodal line in another sample**Same analysis as in Fig. 3 in the main text but on a 3R domain on a different sample.

[1] M. M. Hirschmann, A. Leonhardt, B. Kilic, D. H. Fabini, and A. P. Schnyder, Physical Review Materials **5** (2021), 10.1103/physrevmaterials.5.054202.

[2] W.-Y. He, X. Y. Xu, and K. T. Law, Communications Physics **4**, 66 (2021).

[3] K. Parshukov, Master's thesis, University of Stuttgart, 2023 (unpublished).

[4] A. Leonhardt, M. M. Hirschmann, N. Heinsdorf, X. Wu, D. H. Fabini, and A. P. Schnyder, Phys. Rev. Mater. **5**, 124202 (2021).

[5] M. I. Aroyo, D. Orobengoa, G. de la Flor, E. S. Tasci, J. M. Perez-Mato, and H. Wondratschek, Acta Crystallographica Section A **70**, 126–137 (2014).

[6] W. Moritz and M. A. van Hove, *Surface Structure Determination by LEED and X-rays* (Cambridge University Press, Cambridge, 2022) Chap. 7.

[7] C. Schamper, H. L. Meyerheim, and W. Moritz, Journal of Applied Crystallography **26**, 687 (1993).

[8] E. Vlieg, Journal of Applied Crystallography **30**, 532 (1997).

[9] G. M. Sheldrick, Acta Crystallographica Section C **71**, 3 (2015).

[10] A. Meetsma, G. A. Wiegers, R. J. Haange, and J. L. de Boer, Acta Crystallographica Section A Foundations of Crystallography **45**, 285–291 (1989).

[11] Y. Gotoh, J. Akimoto, and Y. Oosawa, Journal of Alloys and Compounds **270**, 115–118 (1998).

[12] S. C. Abrahams, Acta Crystallographica Section A **25**, 165 (1969).

[13] H. Katzke, P. Tolédano, and W. Depmeier, Physical Review B **69** (2004), 10.1103/physrevb.69.134111.

[14] H. P. Hughes and J. A. Scarfe, Journal of Physics: Condensed Matter **8** (1996), 10.1088/0953-8984/8/10/016.

[15] J. A. Scarfe and H. P. Hughes, Journal of Physics: Condensed Matter **1** (1989), 10.1088/0953-8984/1/38/011.
